# Lack of Association between Recurrent Pregnancy Loss and Inherited Thrombophilia in a Group of Colombian Patients

**DOI:** 10.1155/2012/367823

**Published:** 2012-04-11

**Authors:** Henry Cardona, Serguei A. Castañeda, Wálter Cardona Maya, Leonor Alvarez, Joaquín Gómez, Jorge Gómez, José Torres, Luis Tobón, Gabriel Bedoya, Ángela P. Cadavid

**Affiliations:** ^1^Grupo Reproducción, Universidad de Antioquia, Medellín, Colombia; ^2^Grupo de Estudio en Trombosis, Universidad de Antioquia, Medellín, Colombia; ^3^Departamento de Ginecología y Obstetricia, Universidad de Antioquia and Hospital Universitario San Vicente de Paúl, Medellín, Colombia; ^4^GENMOL, Universidad de Antioquia, Medellín, Colombia

## Abstract

Studies have shown an association between recurrent pregnancy loss and inherited thrombophilia in Caucasian populations, but there is insufficient knowledge concerning triethnic populations such as the Colombian. The aim of this study was to evaluate whether inherited thrombophilia is associated with recurrent pregnancy loss. *Methods*. We conducted a case-control study of 93 patients with recurrent pregnancy loss (cases) and 206 healthy multiparous women (controls) in a Colombian subpopulation. Three single nucleotide polymorphisms (SNPs) markers of the inherited thrombophilias factor V Leiden, prothrombin G20210A, and methylenetetrahydrofolate reductase C677T were genotyped by PCR-RFLP. Activated protein C resistance and plasma levels of antithrombin, protein C, and protein S were also measured. *Results*. The frequency of thrombophilia-associated SNPs, activated protein C resistance, and anticoagulant protein deficiencies, was low overall, except for the methylenetetrahydrofolate reductase C677T SNP. The differences between patients and controls had no statistical significance. *Conclusion*. Our study confirms the low prevalence of inherited thrombophilias in non-Caucasian populations and it is unlikely that the tested thrombophilias play a role in the pathogenesis of recurrent pregnancy loss in this Colombian population.

## 1. Introduction

Pregnancy is an acquired hypercoagulable state and women with a prior tendency to thrombosis may develop clinical symptoms of placental vascular complications such as preeclampsia, intrauterine growth restriction, and fetal death for unknown causes, that impact the maternal-fetal morbidity and mortality [1–4]. Recurrent pregnancy loss is an important obstetric complication with a prevalence of 1–5% [5]. Inherited thrombophilia has been postulated as a cause of recurrent pregnancy loss, although the association between inherited thrombophilia and recurrent pregnancy loss has not been conclusively established. Some studies have demonstrated an association between recurrent pregnancy loss and prothrombotic states rendered by some genetic single nucleotide polymorphisms (SNPs), such as factor V Leiden G1691A (FV Leiden), prothrombin G20210A (FII G20210A), and methylenetetrahydrofolate reductase C677T (MTHFR C677T), and activated protein C resistance (APC resistance) [6–14], whereas others have reported lack of any association [15–19]. In addition, a retrospective cohort study showed that women with deficiencies of antithrombin (AT), protein C (PC), or protein S (PS) have an eightfold increased relative risk of thrombosis during pregnancy compared to controls [20]. 

Most of the studies on thrombophilia and recurrent pregnancy loss have been conducted in Caucasian populations [21, 22]. Therefore, the association between these thrombophilias and recurrent pregnancy loss is almost unknown in triethnic populations such as the Colombian population whose genetic mixture is approximately 70% Caucasian, 15% Amerindian, and 15% African [23, 24]. We conducted this study to evaluate the association between inherited thrombophilia and recurrent pregnancy loss in a group of patients from Colombia.

## 2. Patients and Methods

### 2.1. Study Population

An unmatched case-control study was conducted on 93 patients and 206 healthy women from a province in Northwest Colombia (Antioquia) chosen for convenience. The inclusion criteria for the patient group were a history for recurrent pregnancy loss (three or more miscarriages) and any of the following vascular pregnancy manifestations: one or more second-trimester or later losses, severe or recurrent preeclampsia, intrauterine growth restriction, placental abruption, or otherwise unexplained intrauterine death. The women with recurrent pregnancy loss were selected among more than 3000 patients who had consulted, over the last 20 years, to the Reproduction Group at Universidad de Antioquia in Medellín, Colombia. The control group included 206 healthy women with a history of two or more normal pregnancies, no more than one miscarriage, and no history of any of the criteria for inclusion in the patient group. The sample size estimated for a type I error of 5% and a power of 80% was 300 subjects (2 : 1 ratio between controls and cases). This estimation was based on the following average ratios of SNPs in healthy European populations and their odds ratio for recurrent pregnancy loss: 4.3% of heterozygous FV Leiden with an odds ratio (OR) of 4 [8, 25, 26]; 3% of FII G20210A with OR of 3.7 [8, 26]; 9% of MTHFR C677T with an OR of 3.1 [26–28]. European populations were selected as reference because insufficient information was available about the frequency of inherited thrombophilia in a population with mixed genetic ancestry such as the Colombian. Additionally, deficiencies of AT, PC, and PS were determined in an exploratory manner, as a pilot study, since the sample size was not powered to discriminate differences between patients and controls. This study was approved by the Ethics Committee of the Medical Research Center at the Universidad de Antioquia and all subjects provided informed consent.

### 2.2. Thrombophilia Screening

Blood samples were drawn from women who were not taking any anticoagulant drugs and who were at least 3 months past the last obstetric event to prevent changes in the APC resistance status and plasma levels of coagulation proteins.

Thrombophilia-associated SNPs were genotyped using the methods described by Ridker et al. [29] (FV Leiden), Poort et al. [30] (FII G20210A), and Mandel et al. [31] (MTHFR C677T). The APC resistance status was determined in plasma using a second-generation functional assay based on activated partial thromboplastin time with factor-V-deficient plasma (Test APC RESISTANCE V, Instrumentation Laboratory, Lexington, MA, USA) considering a positive APC resistance when the ratio *a*/*b* was lower than 2 being (a) in the presence, and (b) in the absence of activated PC. The activities of AT, PC, and PS were determined with commercial kits (IL Test Antithrombin, IL Test ProClot, and IL Test Protein S, resp.; Instrumentation Laboratory, Lexington, MA, USA). These proteins were considered deficient when their activity was <50% of their normal functionality.

### 2.3. Statistical Analysis

The sample size was calculated using the software *tamaño de la muestra* (versión 1.1) [32]. The allele and genotype frequencies were compared between patients and controls with a Mantel and Haenzel's chi-square test and one-tail analysis using SPSS 15.0 [33]. The ORs were calculated with 95% Cornfield's confidence intervals (CIs) using Epi 6 Stat Calc [34].

## 3. Results 

Demographics of the women from the case and the control groups are shown in Table 1. In 93 cases and 206 controls, the frequency of thrombophilia-associated SNPs (as shown in Table 2) for both FV Leiden and FII G20210A heterozygous carriers was one patient and one control (1.1% versus 0.5%, resp.; OR 2.25; 95% CI, 0–83). Homozygous carriers of the MTHFR 677T allele were detected in 12 patients and 30 controls (12.9% versus 14.9%, resp.; OR 0.85; 95% CI, 0.4–1.8). APC resistance was found in 5 of 87 patients versus 8 of 187 controls (5.7% versus 4.3%, resp.; OR 1.36; 95% CI, 0.4–4.8) of which only one women in each group was attributable to FV Leiden. Additionally, none of the patients and only two of the controls had <50% AT activity (0 versus 1%, resp.; OR 0; 95%, CI 0–9.2), and 2 of 91 patients versus 3 of 195 controls showed <50% in PC activity (2.2 versus 1.5%, resp.; OR 1.44; 95% CI, 0.2–10.8), as shown in Table 3. Since the determinations of PS levels were nonreproducible in stored samples, these measurements were excluded from analysis in this study. No association was observed between recurrent pregnancy loss and inherited thrombophilia by either SNP genotypes (FV Leiden, FII G20210A, and MTHFR C677T) or functional phenotypes (APC resistance, PC deficiency, and AT deficiency). 

## 4. Discussion 

 The present study is the first to report the frequency of inherited thrombophilia (thrombophilic polymorphisms and functional tests) in a group of Colombian patients with recurrent pregnancy loss. Thrombophilia-associated SNPs such FV Leiden and FII G20210A were very low in both patients and controls, and although the frequency of MTHFR C677T SNPs was high in the patients, differences were not significant. Other inherited thrombophilias such as PC and AT deficiency are not commonly found in patients who had already experienced recurrent pregnancy loss. Only three patients with PC deficiency and no patients with AT deficiency were found among 93 cases. Overall, we found a low frequency of inherited thrombophilia in our patient population unlike Caucasian populations; therefore, it is unlikely that the thrombophilic alterations studied play a role in the pathogenesis of recurrent pregnancy loss in this Latin American population. 

The low frequency of FV Leiden in our study agrees with two previous reports on the Colombian population. Camacho Vanegas et al. tested 150 healthy individuals from Bogota (30–50 years old) using PCR-RFLP and found no evidence of FV Leiden [35]; however, the allelic frequency of the MTHFR C677T SNP was 0.487, one of highest reported in the literature (70/150 heterozygous CT and 38/150 homozygous TT) [35]. The other study was performed by Varela et al. and used PCR-sequence specific oligonucleotide probes in 495 Colombian blood donors from banks in four cities, reporting a prevalence of FV Leiden of 1.9% (7/370) in individuals from Bogota, while it was 0% in 125 individuals from the other three cities (Medellín, Bucaramanga, and Barranquilla) [36]. In addition, the presence of inherited thrombophilia in 100 Colombian patients with deep venous thrombosis and in a control group (*n* = 114) was evaluated with the following results in patients versus controls: 10% versus 0.9% were heterozygous for FV Leiden, 3% versus 0% were heterozygous for FII G20210A (one patient was homozygous), 24% versus 19.3% were homozygous for MTHFR 677T, and 25.2% versus 5.3% had APC resistance (these thrombophilias reached statistical significance, except for the MTHFR polymorphisms) [37]. During this research, the first homozygous family for the G20210A prothrombin polymorphism reported in Latin America, although only some carriers developed venous thrombosis [38]. 

The FV Leiden, as genetic factor involved in the etiology of thrombosis, has motivated research of this abnormality as a risk factor for recurrent pregnancy loss, as reviewed by Krabbendam et al. [39]; however, this association has not been easy to confirm in multiple studies, making it necessary to reevaluate the experimental design and population size. For example, Nurk et al. [40] evaluated FV Leiden in 5874 women and found that this polymorphism is a risk factor for pregnancy complications and correlated with adverse outcomes. On the other hand, some researchers have shown no association between inherited thrombophilia and recurrent pregnancy loss [41–45], possibly because either the only evaluated SNP was MTHFR C677T [43, 44] or the sample sizes were small, that is, 40 patients and 20 controls [42] and 55 women with recurrent pregnancy loss and 50 controls [41]. Additionally, in a Malasyan population, the identification of FV Leiden and FII G20210A in women with recurrent pregnancy loss was also low [46]. 

Our results are in contrast with previous recurrent pregnancy loss studies in Latin American populations. Brazilian women carrying FV Leiden had a 4.9 times higher risk of recurrent pregnancy loss than noncarriers [9], and women heterozygous for FV Leiden from Uruguay had a 5 times higher risk of recurrent pregnancy loss than noncarriers (CI 95%, 1.5–21) [47]. Therefore, our study highlights special genetic characteristics in this triethnic population from Colombia compared to populations with a higher percentage of Caucasians [23, 24]. Our results confirm the differences in the genetic background between Caucasian and non-Caucasian populations, in one extreme populations such as the Japanese that virtually lacks FV Leiden carriers [48] and somewhere in the spectrum of thrombophilia frequency, our triethnic population with low frequency but identifiable thrombophilia-associated SNPs. These results provide evidence of how a genetic component such as polymorphisms in genes associated with thrombosis and its interaction with diverse environmental factors such as food, geographical location, medical resources, public health, and the age of the woman could lead to variable manifestations of complex diseases such as recurrent pregnancy loss. 

In the current study, the wide confidence intervals suggest that the estimated sample size of 300 individuals was insufficient to detect differences between patients with recurrent pregnancy loss and control women for whom the power of the study decreased from an estimated 80% to 30% for detections of an OR of 2.0 with a frequency of 0.5% for FV Leiden in the controls. Nevertheless, our results are valuable because they confirm that the frequency of these inherited thrombophilias in our populations is low; therefore, we believe that there is no justification to recommend testing inherited thrombophilias in the initial evaluation of patients with recurrent pregnancy loss. To detect an association between thrombophilias and recurrent pregnancy loss, it would be necessary to increase considerably the sample size but with unlikely clinical significance.

## Figures and Tables

**Table 1:** Characteristics of patients and controls.

Characteristics	Cases (*n* = 93)	Controls (*n* = 206)
Age, mean ± SD (years)	34.1 ± 0.9	41.6 ± 0.7
Age, range (years)	18–79	23–83
Number of previous pregnancies, mean ± SD	4.4 ± 1.9	2.8 ± 1.3
Number of pregnancy losses, mean ± SD	3.8 ± 1.4	0.1 ± 0.3
Family history of venous thrombosis (%)	25.8	29
Medical history of venous thrombosis (%)	6.5	0
History of severe or recurrent preeclampsia (%)	13.0	0
History of pregnancies with intrauterine growth restriction (%)	1,9	0
History of placental abruption (%)	0.9	0

SD: standard deviation; *n*: number of women

**Table 2:** Genotype frequency of thrombophilias.

SNP	Genotype	Cases, *n* (%)	Controls, *n* (%)	OR (CI 95%)
FV Leiden	GG (normal)	92 (98.9%)	205 (99.5%)	0.4 (0–16.44)
	GA	1 (1.1%)	1 (0.5%)	2.2 (0–83)
	AA	0	0	ND

Total		93 (100%)	206 (100%)	

FII G20210A	GG (normal)	92 (98.9%)	205 (99.5%)	0.4 (0–16.4)
	GA	1 (1.1%)	1 (0.5%)	2.2 (0–83)
	AA	0	0	ND

Total		93 (100%)	206 (100%)	

MTHFR C677T	CC (normal)	38 (40.9%)	93 (45.1%)	0.8 (0.5–1.4)
	CT	43 (46.2%)	83 (40%)	1.3 (0.8–2.1)
	TT	12 (12.9%)	30 (14.9%)	0.8 (0.4–1.8)

Total		93 (100%)	206 (100%)	

SNP: single nucleotide polymorphism; *n*: number of women; OR: odds ratio; CI 95%: Cornfield's 95% confidence interval for the OR; ND: not determined (0% frequency); FV: factor V; FII: prothrombin; MTHFR: methylenetetrahydrofolate reductase.

**Table 3:** Frequency of antithrombin deficiency, protein C deficiency, and activated protein C resistance.

Alteration	Cases, *n* (%)	Controls, *n* (%)	OR (CI 95%)*
AT deficiency (<50%)	0	2 (1%)	0 (0–9.2)
PC deficiency (<50%)	2 (2.2%)	3 (1.5%)	1.44 (0.2–10.8)
APC resistance	5 (5.7%)	8 (4.3%)	1.36 (0.4–4.8)
APC resistance with FV Leiden	1 (20%)	1 (12.5%)	1.75 (0–93)
APC resistance without FV Leiden	4 (80%)	7 (87.5%)	N/A

*n*: number of women; OR: odds ratio; CI 95%: Cornfield's 95% confidence interval for the OR; AT: antithrombin; PC: protein C; APC: activated protein C.

N/A: not applicable.

## References

[1] Gemmati D, Serino ML, Moratelli S, Tognazzo S, Ongaro A, Scapoli GL (2001). Coexistence of factor V G1691A and factor II G20210A gene mutations in a thrombotic family is associated with recurrence and early onset of venous thrombosis. *Haemostasis*.

[2] Rai R, Regan L (2000). Thrombophilia and adverse pregnancy outcome. *Seminars in Reproductive Medicine*.

[3] Stirling Y, Woolf L, North WRS (1984). Haemostasis in normal pregnancy. *Thrombosis and Haemostasis*.

[4] Jivraj S, Anstie B, Cheong YC, Fairlie FM, Laird SM, Li TC (2001). Obstetric and neonatal outcome in women with a history of recurrent miscarriage: a cohort study. *Human Reproduction*.

[5] Christiansen OB, Andersen AMN, Bosch E (2005). Evidence-based investigations and treatments of recurrent pregnancy loss. *Fertility and Sterility*.

[6] Sarig G, Younis JS, Hoffman R, Lanir N, Blumenfeld Z, Brenner B (2002). Thrombophilia is common in women with idiopathic pregnancy loss and is associated with late pregnancy wastage. *Fertility and Sterility*.

[7] Wramsby ML, Sten-Linder M, Bremme K (2000). Primary habitual abortions are associated with high frequency of Factor V Leiden mutation. *Fertility and Sterility*.

[8] Brenner B, Sarig G, Weiner Z, Younis J, Blumenfeld Z, Lanir N (1999). Thrombophilic polymorphisms are common in women with fetal loss without apparent cause. *Thrombosis and Haemostasis*.

[9] Souza SS, Ferriani RA, Pontes AG, Zago MA, Franco RF (1999). Factor V Leiden and factor II G20210A mutations in patients with recurrent abortion. *Human Reproduction*.

[10] Goodman CS, Coulam CB, Jeyendran RS, Acosta VA, Roussev R (2006). Which thrombophilic gene mutations are risk factors for recurrent pregnancy loss?. *American Journal of Reproductive Immunology*.

[11] Foka ZJ, Lambropoulos AF, Saravelos H (2000). Factor V Leiden and prothrombin G20210A mutations, but not methylenetetrahydrofolate reductase C677T, are associated with recurrent miscarriages. *Human Reproduction*.

[12] Kovalevsky G, Gracia CR, Berlin JA, Sammel MD, Barnhart KT (2004). Evaluation of the association between hereditary thrombophilias and recurrent pregnancy loss: a meta-analysis. *Archives of Internal Medicine*.

[13] Pihusch R, Buchholz T, Lohse P (2001). Thrombophilic gene mutations and recurrent spontaneous abortion: prothrombin mutation increases the risk in the first trimester. *American Journal of Reproductive Immunology*.

[14] Rey E, Kahn SR, David M, Shrier I (2003). Thrombophilic disorders and fetal loss: a meta-analysis. *Lancet*.

[15] Rai R, Shlebak A, Cohen H (2001). Factor V leiden and acquired activated protein C resistance among 1000 women with recurrent miscarriage. *Human Reproduction*.

[16] Pauer H-U, Voigt-Tschirschwitz T, Hinney B (2003). Analyzes of three common thrombophilic gene mutations in German women with recurrent abortions. *Acta Obstetricia et Gynecologica Scandinavica*.

[17] Kutteh WH, Park VM, Deitcher SR (1999). Hypercoagulable state mutation analysis in white patients with early first-trimester recurrent pregnancy loss. *Fertility and Sterility*.

[18] Pickering W, Marriott K, Regan L (2001). G20210A prothrombin gene mutation: prevalence in a recurrent miscarriage population. *Clinical and Applied Thrombosis/Hemostasis*.

[19] Carp H, Salomon O, Seidman D, Dardik R, Rosenberg N, Inbal A (2002). Prevalence of genetic markers for thrombophilia in recurrent pregnancy loss. *Human Reproduction*.

[20] Friederich PW, Sanson BJ, Simioni P (1996). Frequency of pregnancy-related venous thromboembolism in anticoagulant factor-deficient women: implications for prophylaxis. *Annals of Internal Medicine*.

[21] Regan L, Rai R (2002). Thrombophilia and pregnancy loss. *Journal of Reproductive Immunology*.

[22] Kupferminc MJ (2005). Thrombophilia and pregnancy. *Current Pharmaceutical Design*.

[23] Bedoya G, Montoya P, García J (2006). Admixture dynamics in Hispanics: a shift in the nuclear genetic ancestry of a South American population isolate. *Proceedings of the National Academy of Sciences of the United States of America*.

[24] Carvajal-Carmona LG, Soto ID, Pineda N (2000). Strong Amerind/white sex bias and a possible Sephardic contribution among the founders of a population in Northwest Colombia. *American Journal of Human Genetics*.

[25] Herrmann FH, Koesling M, Schröder W (1997). Prevalence of factor V Leiden mutation in various populations. *Genetic Epidemiology*.

[26] Kupferminc MJ, Eldor A, Steinman N (1999). Increased frequency of genetic thrombophilia in women with complications of pregnancy. *New England Journal of Medicine*.

[27] Murphy RP, Donoghue C, Nallen RJ (2000). Prospective evaluation of the risk conferred by factor V Leiden and thermolabile methylenetetrahydrofolate reductase polymorphisms in pregnancy. *Arteriosclerosis, Thrombosis, and Vascular Biology*.

[28] Raziel A, Kornberg Y, Friedler S, Schachter M, Sela BA, Ron-El R (2001). Hypercoagulable thrombophilic defects and hyperhomocysteinemia in patients with recurrent pregnancy loss. *American Journal of Reproductive Immunology*.

[29] Ridker PM, Miletich JP, Hennekens CH, Buring JE (1997). Ethnic distribution of factor V Leiden in 4047 men and women: implications for venous thromboembolism screening. *Journal of the American Medical Association*.

[30] Poort SR, Rosendaal FR, Reitsma PH, Bertina RM (1996). A common genetic variation in the 3′-untranslated region of the prothrombin gene is associated with elevated plasma prothrombin levels and an increase in venous thrombosis. *Blood*.

[31] Mandel H, Brenner B, Berant M (1996). Coexistence of hereditary homocystinuria and Factor V Leiden—effect on thrombosis. *New England Journal of Medicine*.

[32] Pérez A, Rodríguez N, Gil J, Ramírez G Tamaño de muestra. In. (Bogotá, Pontificia Universidad Javeriana. Facultad de Medicina. Unidad de Epidemiología Clínica y Bioestadística.), p Programa sistematizado para el cálculo del tamaño de la muestra y el poder en diseños de investigación.

[34] Dean AG, Dean JA, Coulombier D (1994). EPI 6 STAT CALC. *Epi Info Version 6: A Word Processing, Database, and Statistics Program for Epidemiology on Microcomputers*.

[35] Camacho Vanegas O, Giusti B, Restrepo Fernandez CM, Abbate R, Pepe G (1998). Frequency of factor V (FV) Leiden and C677T methylenetetrahydrofolate reductase (MTHFR) mutations in Colombians. *Thrombosis and Haemostasis*.

[36] Varela A, Bilbao A, Garcia CF (2000). Prevalencia de la mutacion del factor V de la coagulacion (factor V Leiden) en donantes de banco de sangre en cuatro ciudades colombianas. *Acta Médica Colombiana*.

[37] Torres Hernández JD, Cardona H, Álvarez L (2006). Inherited thrombophilia is associated with deep vein thrombosis in a Colombian population. *American Journal of Hematology*.

[38] Roman-Gonzalez A, Cardona H, Cardona-Maya W (2009). The first homozygous family for prothrombin G20210A polymorphism reported in Latin America. *Clinical and Applied Thrombosis/Hemostasis*.

[39] Krabbendam I, Franx A, Bots ML, Fijnheer R, Bruinse HW (2005). Thrombophilias and recurrent pregnancy loss: a critical appraisal of the literature. *European Journal of Obstetrics Gynecology and Reproductive Biology*.

[40] Nurk E, Tell GS, Refsum H, Ueland PM, Vollset SE (2006). Factor V Leiden, pregnancy complications and adverse outcomes. The Hordaland Homocysteine Study. *Quarterly Journal of Medicine*.

[41] Balasch J, Reverter JC, Fábregues F (1997). First-trimester repeated abortion is not associated with activated protein C resistance. *Human Reproduction*.

[42] Dizon-Townson DS, Kinney S, Ware Branch D, Ward K (1997). The factor V Leiden mutation is not a common cause of recurrent miscarriage. *Journal of Reproductive Immunology*.

[43] Holmes ZR, Regan L, Chilcott I, Cohen H (1999). The C677T MTHFR gene mutation is not predictive of risk for recurrent fetal loss. *British Journal of Haematology*.

[44] Makino A, Nakanishi T, Sugiura-Ogasawara M, Ozaki Y, Suzumori N, Suzumori K (2004). No association of C677T methylenetetrahydrofolate reductase and an endothelial nitric oxide synthase polymorphism with recurrent pregnancy loss. *American Journal of Reproductive Immunology*.

[45] Pauer HU, Neesen J, Hinney B (1998). Factor V Leiden and its relevance in patients with recurrent abortions [8]. *American Journal of Obstetrics and Gynecology*.

[46] Ayadurai T, Muniandy S, Omar SZ (2009). Thrombophilia investigation in Malaysian women with recurrent pregnancy loss. *Journal of Obstetrics and Gynaecology Research*.

[47] Otero AM, Pou Ferrari R, Pons E (2004). Trombofilia y pérdida recurrente de embarazo. *Revista Médica del Uruguay*.

[48] Yamada H, Kato EH, Kobashi G (2001). Recurrent pregnancy loss: etiology of thrombophilia. *Seminars in Thrombosis and Hemostasis*.

